# Management of penile prosthesis complications: a case series and review of current surgical strategies

**DOI:** 10.11604/pamj.2025.50.34.45903

**Published:** 2025-01-27

**Authors:** Moussaab Rachid, Hamza Rais, Ghassane El Omri, Younes Houry, Abdeljalil Heddat

**Affiliations:** 1 Department of Urology, Cheikh Khalifa International University Hospital, Mohammed VI University of Health Sciences (*UM6SS*), Casablanca, Morocco

**Keywords:** Penile implants, semirigid penile prostheses, inflatable penile prostheses

## Abstract

Penile prostheses offer a definitive solution for organic erectile dysfunction, particularly in cases where medical therapies are ineffective or contraindicated. Despite advancements in surgical techniques and infection-resistant materials, complications still arise. This article presents a case series of three patients experiencing different penile prosthesis complications, including severe penile pain, perineal infection, and prosthesis displacement. Management strategies included surgical removal and replacement of prostheses, administration of antibiotics, and careful post-operative monitoring. Key complications discussed include infections, erosion, mechanical malfunctions, and persistent pain. Effective management involves strict aseptic techniques, patient education, and prompt surgical intervention when necessary. This study underscores the importance of meticulous surgical practice and ongoing patient care to mitigate complications and improve outcomes for penile prosthesis recipients.

## Introduction

Penile prostheses provide a definitive solution for managing organic erectile dysfunction in patients who either cannot undergo medical treatments, such as phosphodiesterase inhibitors or have not responded to conservative approaches [1]. Recent advancements in surgical techniques and infection-resistant materials have significantly lowered the risks associated with surgery. However, complications, although less common, can still occur and require timely identification and management.

## Methods

This case series involves three patients who presented with various complications related to penile prostheses, all of which were managed surgically. The study was conducted from June 2023 to February 2024 at the Cheikh Khalifa International University Hospital in Casablanca, Morocco. The patients included in this study were all male individuals who were admitted through the emergency department due to complications associated with penile prostheses that had been implanted at external facilities. The complications encountered included prosthesis extrusion, mechanical malfunction, and localized infections. Upon admission, each patient underwent a comprehensive evaluation consisting of a detailed medical history, including prior medical and surgical conditions as well as information regarding the type of penile prosthesis implanted. A clinical examination followed this to assess the general condition, measure vital signs, and thoroughly inspect the genital area. Paraclinical investigations, including laboratory tests and penile Doppler ultrasonography, were performed to assess the nature of the complications further.

Surgical interventions were tailored according to the specific type of complication. In cases of prosthesis extrusion, manual extraction was performed, followed by urethral repair when necessary. Broad-spectrum empirical antibiotic therapy, consisting of third-generation cephalosporins and aminoglycosides, was administered before and after surgery to prevent or manage infections. In cases requiring prosthesis replacement, a new malleable prosthesis was implanted, with adjustments made to the original incision site to minimize the risk of recurrence. Postoperatively, all patients were hospitalized for an average of 5 to 7 days. The postoperative care regimen included close monitoring of vital signs, daily assessment of the surgical wound, and laboratory follow-up with C-reactive protein (CRP) and leukocyte counts to detect potential infections. Follow-up consultations were scheduled at one, three, and six months post-surgery to evaluate functional recovery and identify any late-onset complications. Data for this study were collected retrospectively from electronic medical records and analyzed to assess the types of complications, the management approaches employed, and the associated surgical outcomes.

## Results

**Figure 1:**
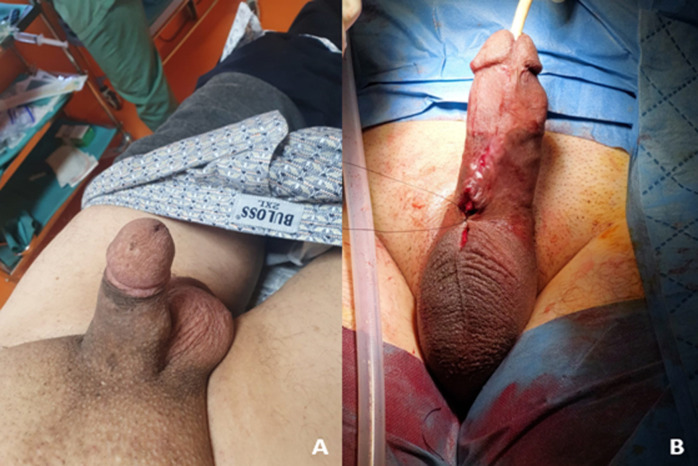
A) unilateral deviation noted in the corpus cavernosum; B) penile prosthesis reimplantation

**Case 1**: a 65-year-old male patient presented to the emergency department complaining of excruciating pain localized to the penile region for the past 48 hours. He reported a medical history significant for diabetes mellitus managed with oral antidiabetic medications. The pain was described as severe and constant, without alleviation with over-the-counter analgesics. Additionally, the patient reported a history of erectile dysfunction refractory to medical treatment, which necessitated the placement of a Rigicon penile prosthesis approximately two years ago. Upon examination, the patient appeared in a preserved general condition, alert, and oriented. Examination of the genitalia revealed an oedematous appearance of the penis with no evident signs of erythema or ulceration. Palpation elicited tenderness throughout the penile shaft, with a palpable unilateral deviation noted in the corpus cavernosum (Figure 1 A). Furthermore, there was an asymmetrical aspect observed between the two cavernous bodies, suggestive of Peyronie's disease or penile ischemia. The patient's medical records indicate a history of Rigicon penile prosthesis placement for erectile dysfunction refractory to medical treatment. The patient's presentation, coupled with his medical history, suggests a potential complication related to the penile prosthesis, such as implant malfunction or penile ischemia. Given the failure of medical treatment for erectile dysfunction and the subsequent placement of a Rigicon penile prosthesis, consideration must be given to the possibility of prosthesis-related complications contributing to the current symptoms. Immediate measures will include pain management with intravenous analgesics and close monitoring of vital signs. Laboratory investigations, including a complete blood count, comprehensive metabolic panel, and serum markers for penile ischemia, will be obtained. Imaging studies such as penile duplex ultrasonography may be indicated to assess vascular integrity and detect any structural abnormalities related to the prosthesis. Consultation with a urologist will be sought for further evaluation and management. If prosthesis-related complications are confirmed, surgical intervention may be necessary, including removal and replacement of the penile prosthesis. Given the complexity of the case, consideration will be given to changing the Rigicon prosthesis to a symmetrical Genesis prosthesis and adapting the measurements to optimize outcomes and minimize the risk of recurrent complications (Figure 1 B).

**Figure 2:**
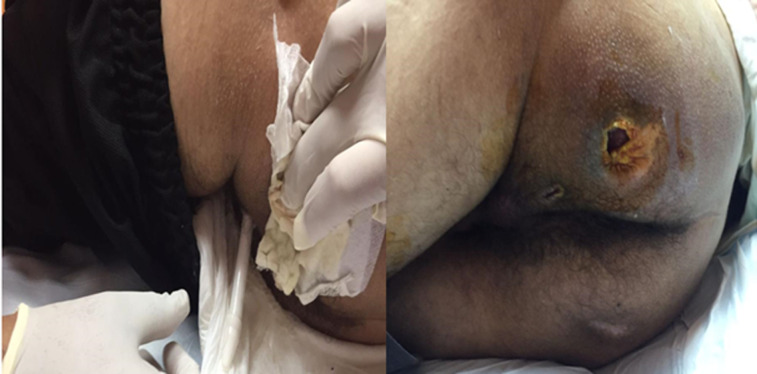
extended penile prosthesis from the gluteal region

**Case 2**: a 37-year-old male patient with a history of polytrauma four years ago resulting in paraplegia presented to the urology clinic with complaints of perineal pain and issues related to a recently placed malleable penile prosthesis. The patient reported undergoing the placement of malleable prostheses approximately seven months before admission. He described localized discomfort in the perineal region, which worsened over the past few days. Upon examination, the patient appeared alert and oriented, with a preserved general condition. Inspection of the perineal area revealed erythema and induration surrounding the site of the prosthesis, extending from the gluteal region (Figure 2). Palpation elicited tenderness and warmth over the affected area. A point of care was noted at the site of the prosthesis issue, indicative of tissue breakdown and potential infection. There were no signs of systemic inflammation, and the patient remained afebrile. The patient's presentation is consistent with local complications related to the malleable penile prosthesis, likely secondary to pressure necrosis from prolonged contact with the gluteal area. The development of perineal pain and tissue breakdown raises concerns for infection and warrants prompt intervention to prevent further complications. Immediate measures will include the removal of the penile prosthesis to alleviate pressure on the affected area and mitigate the risk of worsening tissue damage (Figure 2). Antibiotic therapy with broad-spectrum coverage, including ceftriaxone and ciprofloxacin, will be initiated to address potential infection. Local wound care, including regular cleansing and dressing changes, will be implemented to promote healing and prevent secondary infection. Close monitoring of the patient's clinical status, including vital signs and wound appearance, will guide ongoing management. Additionally, rehabilitation services will be involved to address any functional limitations resulting from the prosthesis removal and optimize the patient's overall recovery.

**Figure 3:**
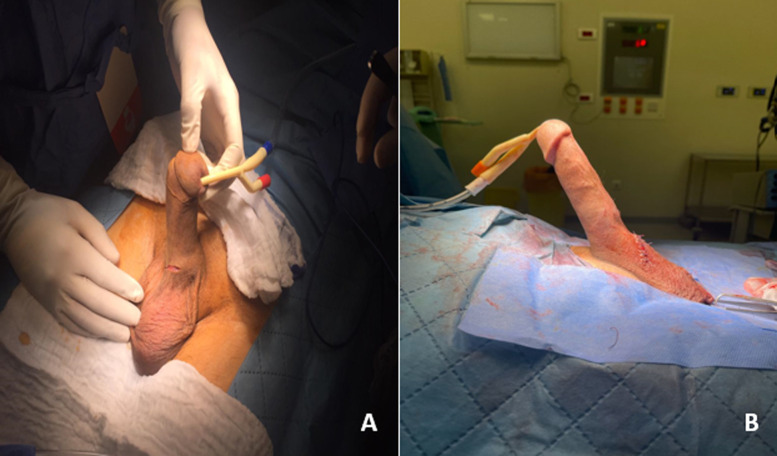
unilateral displacement of the penile prosthesis; A) with erosion noted along the cavernous body; B) the surgical removal

**Case 3**: a 55-year-old male patient with a history of uncontrolled hypertension despite medical treatment presented to the urology clinic with complaints related to a malleable penile prosthesis placed approximately twelve months ago. The patient reported persistent erectile dysfunction refractory to medical therapy, prompting the placement of the prosthesis. He described the recent onset of penile pain and discomfort, which worsened over the past few days. Upon examination, the patient appeared alert but displayed signs of discomfort. His blood pressure readings were indicative of uncontrolled hypertension. Examination of the genitalia revealed unilateral displacement of the penile prosthesis, with erosion noted along the cavernous body (Figure 3 A). The affected area appeared erythematous and tender to palpation, suggestive of local inflammation and possible infection. The patient's presentation is consistent with complications related to the malleable penile prosthesis, characterized by unilateral displacement and erosion of the cavernous body. The underlying uncontrolled hypertension may have contributed to the development of vascular compromise and subsequent prosthesis-related complications. Immediate measures will include addressing the patient's hypertensive crisis with antihypertensive medications to stabilize blood pressure. Removal of the displaced penile prosthesis will be performed to prevent further damage and mitigate the risk of infection (Figure 3 B). Antibiotic therapy will be initiated to treat any existing infection and prevent its spread. Close monitoring of the patient's blood pressure and clinical status will guide ongoing management. Additionally, consideration will be given to alternative treatment modalities for erectile dysfunction, given the failure of both medical therapy and prosthesis placement. Referral to a hypertension specialist may also be warranted to optimize blood pressure control and reduce the risk of future complications.

## Discussion

Penile prosthesis implantation remains a definitive treatment option for patients with erectile dysfunction (ED) refractory to medical therapy. Despite advances in surgical techniques and the development of infection-resistant materials, complications still occur, as illustrated by the cases in our study. The most common complications include prosthesis erosion, extrusion, mechanical failure, and infections [2]. Our experience with these three cases underscores the complexity of managing penile prosthesis complications and highlights the need for tailored surgical approaches. In our series, complications included prosthesis extrusion, mechanical malfunction, and infections-findings consistent with those reported in the literature [3,4]. For instance, Wilson *et al*. [5]. noted that mechanical failure and infection remain among the most common complications, even with the latest prosthetic models [5]. The presence of infections often necessitates device removal, which significantly impacts patient satisfaction and increases healthcare costs [6]. In Case 1, the patient presented with severe pain due to mechanical dysfunction, which was suspected to be related to penile ischemia. This highlights the need for thorough diagnostic evaluations, including Doppler ultrasonography, to assess prosthesis integrity and penile blood flow [7]. The choice to replace the malfunctioning Rigicon prosthesis with a Genesis model was made to optimize mechanical reliability, as supported by studies showing that three-piece inflatable prostheses offer better patient satisfaction due to their closer mimicry of natural erections [3,8].

Case 2 presented a unique challenge, with a paraplegic patient experiencing pressure necrosis due to prolonged contact between the malleable prosthesis and surrounding tissues. This complication is often exacerbated in patients with reduced mobility and sensation [9]. As suggested by Kaspar and Henkel [10], careful patient selection and counseling are essential to minimize the risk of complications, particularly in patients with comorbidities such as spinal cord injuries [10]. The patient in Case 3 exhibited prosthesis displacement and erosion in the context of poorly controlled hypertension. Studies indicate that vascular comorbidities, such as hypertension and diabetes, are significant risk factors for prosthesis complications [11]. Effective management of these underlying conditions is critical to reducing the incidence of postoperative complications. Despite these challenges, our postoperative outcomes were favorable, with all patients achieving satisfactory recovery. The use of broad-spectrum antibiotic prophylaxis was effective in preventing severe infections. However, as Gross *et al*. [12] noted, the presence of biofilms on explanted devices underscores the importance of perioperative infection control measures [12]. Overall, our study highlights the importance of individualized patient management in penile prosthesis surgery. A multidisciplinary approach, including thorough preoperative evaluation, meticulous surgical technique, and comprehensive postoperative follow-up, is essential to optimize patient outcomes and reduce complications. Further studies with larger patient cohorts are needed to validate these findings and to explore newer strategies for managing complex cases, particularly in patients with significant comorbidities.

## Conclusion

Penile prosthesis implantation is a highly successful treatment for erectile dysfunction, typically used when other therapies have failed. Advances in surgical tools and biomedical engineering have significantly reduced complications and the need for revision surgery. However, urologists must be knowledgeable about various devices and adhere strictly to sterile techniques to ensure successful outcomes.

### What is known about this topic

- Penile prosthesis implantation is a well-established treatment for erectile dysfunction refractory to medical therapy;
- Despite advances in surgical techniques and infection-resistant materials, complications such as prosthesis erosion, mechanical failure, and infections still occur;
- Proper patient selection, meticulous surgical techniques, and comprehensive postoperative care are critical for reducing complications and improving outcomes.

### What this study adds

- This case series highlights real-world challenges and management strategies for complications related to penile prostheses, emphasizing tailored surgical approaches;
- The study underscores the importance of immediate intervention and individualized treatment plans for complex cases, particularly in patients with comorbidities;
- It provides practical insights into the decision-making process for prosthesis replacement, demonstrating how tailored solutions can optimize patient outcomes and satisfaction.

## References

[1] Wespes E, Amar E, Hatzichristou D, Hatzimouratidis K, Montorsi F, Pryor J (2006). EAU Guidelines on erectile dysfunction: an update. Eur Urol.

[2] Scherzer ND, Dick B, Gabrielson AT, Alzweri LM, Hellstrom WJG (2019). Penile Prosthesis Complications: Planning, Prevention, and Decision Making. Sex Med Rev.

[3] Topuz B, Ebiloğlu T, Zor M, Kaya E, Sarğkaya S, Emrah Coğuplugil A (2021). Penile prosthesis implantation: A single center 25 years of experience. Prog Urol.

[4] Pozza D, Marcantonio A, Mosca A, Pozza C (2020). Penile prosthesis and complications: Results from 577 implants. Arch Ital Urol Androl.

[5] Wilson SK, Delk JR, Salem EA, Cleves MA (2007). Long-term survival of inflatable penile prostheses: single surgical group experience with 2,384 first-time implants spanning two decades. J Sex Med.

[6] Hartman RP, Kawashima A, Takahashi N, LeRoy AJ, King BF (2016). Inflatable penile prosthesis (IPP): diagnosis of complications. Abdom Radiol (NY).

[7] Hernández JC, Trost L, Köhler T, Ring J, Traweek R, Alom M (2019). Emerging Complications Following Alternative Reservoir Placement during Inflatable Penile Prosthesis Placement: A 5-Year Multi-Institutional Experience. J Urol.

[8] Çayan S, Aşcı R, Efesoy O, Bolat MS, Akbay E, Yaman Ö (2019). Comparison of Long-Term Results and Couples´ Satisfaction with Penile Implant Types and Brands: Lessons Learned From 883 Patients With Erectile Dysfunction Who Underwent Penile Prosthesis Implantation. J Sex Med.

[9] Madiraju SK, Wallen JJ, Rydelek SP, Carrion RE, Perito PE, Hakky TS (2019). Biomechanical Studies of the Inflatable Penile Prosthesis: A Review. Sex Med Rev.

[10] Kaspar C, Henkel A (2021). Penile prosthesis. Urologe A.

[11] Ji YS, Ko YH, Song PH, Moon KH (2015). Long-term survival and patient satisfaction with inflatable penile prosthesis for the treatment of erectile dysfunction. Korean J Urol.

[12] Gross MS, Phillips EA, Carrasquillo RJ, Thornton A, Greenfield JM, Levine LA (2017). Multicenter Investigation of the Micro-Organisms Involved in Penile Prosthesis Infection: An Analysis of the Efficacy of the AUA and EAU Guidelines for Penile Prosthesis Prophylaxis. J Sex Med.

